# A 4-Week Electronic-Mentoring Employment Intervention for Youth With Physical Disabilities: Pilot Randomized Controlled Trial

**DOI:** 10.2196/12653

**Published:** 2019-04-24

**Authors:** Sally Lindsay, Elaine Cagliostro, Jennifer Stinson, Joanne Leck

**Affiliations:** 1 Department of Occupational Science and Occupational Therapy University of Toronto and Bloorview Research Institute Holland Bloorview Kids Rehabilitation Hospital Toronto, ON Canada; 2 Bloorview Research Institute Holland Bloorview Kids Rehabilitation Hospital Toronto, ON Canada; 3 Lawrence S Bloomberg Faculty of Nursing Hospital for Sick Children University of Toronto Toronto, ON Canada; 4 Telfer School of Management University of Ottawa Toronto, ON Canada

**Keywords:** social support, mentor, youth, rehabilitation, occupational therapy

## Abstract

**Background:**

Youth with disabilities are more likely to live in poverty and be unemployed compared with youth without disabilities. Such trends are often a result of a lack of support, inaccessible jobs, environmental barriers, and discriminatory attitudes toward people with disabilities. Youth with disabilities also face barriers in accessing vocational preparation programs. One encouraging way that could help address challenges that youth encounter is by providing support through electronic mentoring (e-mentoring).

**Objective:**

The objective of this study was to assess the feasibility of a 4-week Web-based peer e-mentoring employment intervention for youth with physical disabilities.

**Methods:**

We conducted a pilot randomized controlled trial (RCT) to evaluate our intervention, *Empowering youth towards employment*. Participants included youth aged 15 to 25 years who were randomly assigned to an experimental (mentored) or control (nonmentored) group. Our intervention involved having trained youth mentors (ie, near peers who also had a disability) lead Web-based discussion forums while offering peer support and resources, which involved 12 modules (3 topics a week for 4 weeks). Primary outcomes focused on implementation (ie, feasibility and acceptability), whereas secondary outcomes focused on effectiveness (ie, measures of self-determination, career maturity, and social support).

**Results:**

A total of 28 youth (mean age 19.62, SD 3.53; 14/28, 50% female) completed the RCT in 3 intervention groups and 2 control groups (intervention n=18, control n=10). Participants reported satisfaction with the program and that it was feasible and acceptable. Youth’s mean engagement level with the program was 6.44 (SD 2.33) for the experimental group and 5.56 (SD 3.53) for controls. Participants in the intervention group did not demonstrate any significant improvements in social support, career maturity, or self-determination compared with those in the control group. No adverse events were reported.

**Conclusions:**

The *Empowering youth towards employment* e-mentoring intervention needs further testing with a larger sample and different length of formats to understand how it may have an impact on employment outcomes for youth with disabilities.

**Trial Registration:**

ClinicalTrials.gov NCT02522507; https://clinicaltrials.gov/ct2/show/NCT02522507 (Archived by WebCite at http://www.webcitation.org/77a3T4qrE)

## Introduction

### Background

There is a growing literature highlighting the benefits of having a diverse workforce and, particularly, hiring people with disabilities [1]. Although there have been many encouraging improvements in this area, people with disabilities, and particularly youth, continue to experience lower employment rates compared with youth without disabilities. For example, the employment rate for youth in Canada aged 20 to 24 years with a severe disability is 35% and youth with a mild or moderate disability is 57% compared with 87% of youth without disabilities [2]. A similar pattern exists for youth aged 15 to 19 years where 40% of those with disabilities are employed compared with 51% of youth without disabilities [3]. Gaining employment skills is important because engagement in paid employment is a social determinant of health that is linked with improving independence and quality of life [4-6]. Many youth with disabilities would like to work and are capable of doing so but encounter many barriers in preparing for and finding employment [7-9]. Even more concerning is that 34% of youth with disabilities, aged 16 to 24 years, are neither working nor in school [10].

Employment preparation programs are often not designed to meet the needs of youth with physical disabilities [6,11]. Although some programs exist, they are mostly focused on youth with developmental or intellectual disabilities. Youth with physical disabilities arguably encounter different needs and challenges in terms of social development and role functioning [5,12]. A recent systematic review focusing on employment preparation programs for youth with physical disabilities highlighted that there is very limited evidence-based programming for youth [11]. Of the handful of studies in this area, youth with disabilities showed promise with improvements in self-confidence, goal setting, and knowledge of career options [11]. Given the various challenges that youth with disabilities encounter, more efforts are needed to help youth prepare for and engage in meaningful employment [7].

Mentoring is a potential way to strengthen the inclusion of youth with disabilities in employment while also offering meaningful social support [13-15]. A mentor refers to someone who is more experienced, acts as a role model, and shares experiences with a less experienced individual. Research shows that mentoring relationships have potential to offer informational, practical, and emotional support; self-determination; quality of life; and career development goals [13,16-21]. There is a small but growing literature on mentoring programs for youth with disabilities, most of which has focused on traditional face-to-face mentoring. A main challenge with this type of mentoring is that it is often difficult to find and access mentors [13]. Therefore, a potential advantage of electronic mentoring (e-mentoring) is that it is in a format that allows for flexibility in matching a mentor with a mentee and also has asynchronous communication [22]. E-mentoring can help to overcome inequities and provide opportunities to underserved groups such as youth with disabilities [14,15]. Given that Web-based platforms can influence learning and behavior change [23-25], they may offer a promising way to help youth with disabilities to learn essential employment preparation skills. Our intervention aims to strengthen employment readiness skills of youth with disabilities, including their self-determination, career maturity, and social support, all which can improve employment outcomes [13,15,26-28].

### Objectives

Our objective was to assess the feasibility (ie, pilot randomized controlled trial [RCT]) of a 4-week e-mentoring intervention focused on youth with physical disabilities for improving self-determination, career maturity, and social support compared with controls.

## Methods

### Design

We used a pilot RCT with an embedded qualitative design [29] to assess the feasibility of the *Empowering youth towards employment* (ie, 4-week e-mentoring) intervention for youth with physical disabilities. We followed the Medical Research Council Framework for the development and evaluation of RCTs to guide our design [30]. We focused on the development and feasibility phase to ascertain the theoretical foundations and to assess the feasibility of intervention components [6,30]. The intervention group received mentorship from trained youth mentors who guided them through 12 module topics in a Web-based forum [6]. The control group only had access to the Web-based modules and did not receive mentorship.

The content of our intervention was based on several systematic reviews conducted by our team focusing on employment preparation interventions for youth with physical disabilities [11], improving the inclusion of people with disabilities in the workforce [31], and best practices of peer mentorship for improving employment outcomes for youth with disabilities [13]. Needs assessments with youth who have a physical disability also informed the types of informational and social support they look for in a Web-based format [12,32].

### Sample and Recruitment

We recruited youth from a pediatric rehabilitation hospital from April 2017 to August 2018. A researcher sent invitation letters to potentially eligible participants meeting the following inclusion criteria: able to read and write in English, aged 15 to 25 years with a physical disability, have access to a computing device with internet access, currently enrolled in or recently completed a high school diploma in the applied or academic stream, and have no paid work experience [6]. We define disability as an impairment, activity limitation, or participation restriction whereby a disability and functioning are shaped by interactions between health conditions and contextual factors [33]. We excluded youth who had recently completed or who were currently participating in employment preparation or peer support intervention [6].

**Figure 1:**
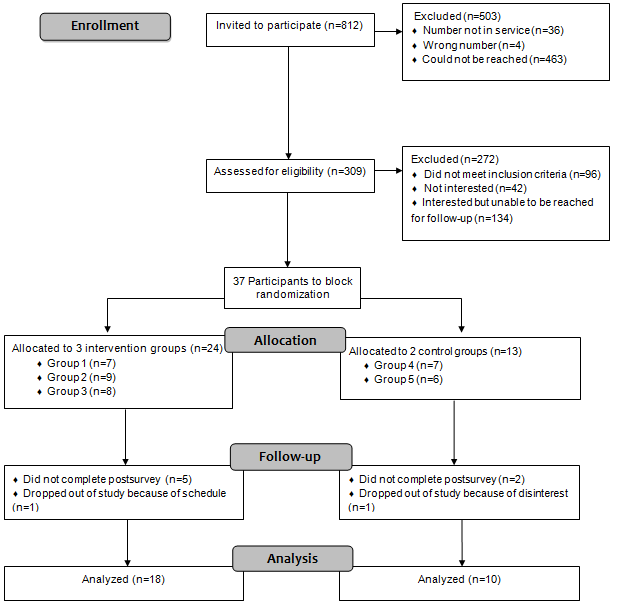
CONSORT flow diagram.

Our aim was to achieve a sample of approximately 80 participants (40 experimental and 40 control) [6] with an alpha of .05 and power of 80% based on the guidelines of Cohen [34] and Hertzog [35]. A total of 37 participants met the inclusion criteria and provided written consent. We used a block size of up to 10 participants who were randomly assigned into an experimental (ie, mentored) or control group (nonmentored; see Figure 1 for trial schema). We ran 5 different groups in the summers of 2017 and 2018, of which 3 were experimental and 2 were control. A total of 7 participants (5 experimental and 2 control) did not complete the postsurvey, and 2 participants dropped out of the study before completion. Moreover, 28 participants completed the intervention and postsurveys (18 experimental and 10 controls).

### Setting

Youth received access to a password-protected area of a website, *AbilityOnline*, a safe forum for youth with disabilities.

### Peer Mentors

Each intervention group had a paid mentor including youth (ie, near peers) who had lived experience with a physical disability, were currently enrolled in postsecondary education, and had completed a 3-day Youth Peer Mentor training program held at a pediatric rehabilitation hospital [36]. A total of 2 of the experimental groups had 1 male and 1 female mentor who led the discussions, and 1 group had 1 male mentor. We also held project-specific training sessions that taught mentors about active listening, perspective taking, confidentiality, maintaining boundaries, positive role modeling, and trust building [6]. We instructed the mentors to introduce the topics in the same order and to respond in a supportive and positive means [6].

### Procedures and Randomization

Ethics approval was obtained from a pediatric hospital and a university. A research assistant screened all potential participants for inclusion and obtained written informed consent before including them in the project. After participants consented, they were randomized into an experimental or control group, with up to 10 participants per group (see Figure 1) [6,37]. A researcher then emailed the presurvey to each participant via *REDCap*. She then informed them of their group assignment and gave them instructions on how to log in to the project website.

#### Intervention (Experimental Group)

The *Empowering youth towards employment* (ie, 4-week e-mentor) intervention was developed to provide social and informational support regarding employment preparation for youth with disabilities [6]. The evidence-informed content [11,13] was cocreated with a knowledge user advisory group consisting of youth with disabilities and parents [6]. The content involved 12 modules where 3 different topics were delivered each week for over 4 weeks. The topics included the following: introduction and goal setting, life skills, managing disability at work, family role in supporting employment, aspirations and expectations, volunteerism, finding a job, social networking and community resources, preparing for job interviews, learning from professionals with disabilities, career pathways and transitions, and referrals [6]. The modules included interactive materials and resources that youth could go through on their own and at their own pace. The experimental group had access to 1 or 2 trained peer mentors through a password-protected Web-based (asynchronous) discussion forum [6]. Each module topic was led by a trained mentor and held in a group-based format, involving up to 10 youth participants and 1 to 2 mentors [6]. The youth mentors used a script to introduce each topic in the same way; however, they were encouraged to share their own relevant experiences. An earlier pilot 12-week version of this intervention is reported elsewhere [6,38]. On the basis of participant feedback that the original format of the program was too long, we condensed the format from 1 weekly topic for 12 weeks to 3 topics per week over 4 weeks [38].

#### Control Group

Participants in the control group (up to 10 per group) were given access to a separate password-protected area of the Web-based forum where they could see the modules but did not receive peer mentorship. Each module topic was posted by a researcher with the same timing as the experimental group, but they did not reply to posts. Participants could interact with other participants in the control group through the discussion forum; however, this was not moderated [6].

### Outcome Measures

Primary outcomes of this study focused on implementation of the 4-week Web-based peer mentoring intervention as measured by recruitment and withdrawal rates, adherence with the intervention (ie, length of time online and number of times logged in), proportion of completed questionnaires, and engagement and satisfaction (ie, self-rated engagement in the study, whether they would recommend the program to others, and open-ended questions to assess satisfaction) [6].

Secondary outcome measures of this study focused on the preliminary estimates of effectiveness of the 4-week Web-based mentoring intervention. In our pre-post surveys, we collected demographic information and the following standardized measures: career maturity inventory attitude scale [39,40], multidimensional scale of perceived social support [41], and self-determination [42]. Each of these measures have been used for youth with disabilities and have good test-retest reliability, internal consistency, and construct-related and criterion validity [40,42,43]. The *career maturity inventory attitude scale* is a 24-item scale including agree and disagree items relating to career decision making (orientation, involvement, independence, compromise, and decisiveness) [26,40]. The *multidimensional scale of perceived social support* is a 12-item questionnaire that captures perceived support from several sources [41]. The scores are summed for a total score where a higher score indicates higher perceived social support [41]. Finally, the *Arc’s self-determination* [42] is a self-report measure for adolescents with disabilities, with subscales on autonomy, acting on the basis of preferences, abilities, postschool directions, goal setting, and task performance.

### Data Analysis

We analyzed the data using IBM SPSS, version 25. We used descriptive statistics to describe sample characteristics at baseline using means and SD for continuous variables and frequencies for categorical variables. We performed *t* tests to compare the baseline characteristics between the experimental and the control groups, separate analyses for each outcome. Analyses were conducted using an intent-to-treat approach. Linear regression models were used to test the intervention effects on outcome measures using an analysis of covariance with posttreatment measures compared between groups using baseline scores as covariates. A level of .05 was used as the criterion for statistical significance. The Holm sequential correction technique was used to control for type I error. We calculated effect sizes using Cohen *d* where 0.2 indicates a small effect, 0.5 medium effect, and 0.8 large effect.

Qualitative data (ie, open-ended survey questions) were analyzed thematically [44] to explore reasons for their satisfaction or dissatisfaction with the program and areas for improvement.

## Results

### Sample Characteristics

A total of 28 youth aged 15 to 25 years (mean age 19.62, SD 3.53; 14/28, 50% female) completed the Web-based program, split between the intervention and control groups. In addition, 12 youth had cerebral palsy, 6 with Duchenne muscular dystrophy or other neuromuscular disorder, 7 with spina bifida, and 3 with other (nonspecified) physical disabilities. Moreover, 22 of the 28 youth (78%) used an assistive or mobility device, and 19 out of 28 youth (68%) were currently enrolled in school. There were no significant differences in demographic variables or comfort with computers and discussion forums between the experimental and control groups at baseline (see Tables 1 and 2).

**Table 1:** Demographic characteristics of participants and mentors.

Demographics		Participants		Mentors (n=3)
		Experimental (n=18)	Control (n=10)	
Age (years), mean (SD)		19.77 (3.49)	19.4 (3.56)	22 (2.64)
**Sex, n (%)**				
	Male	8 (44)	6 (60)	2 (67)
	Female	10 (56)	4 (40)	1 (33)
**Disability type, n (%)**				
	Cerebral palsy	6 (33)	6 (60)	0
	Duchenne muscular dystrophy and neuromuscular	3 (17)	3 (30)	0
	Spina bifida	6 (33)	1 (10)	0
	Other physical disability	3 (17)	0	3 (100)
Use an assistive/mobility device, n (%)		15 (83)	7 (70)	3 (100)
Currently enrolled in school, n (%)		11 (61)	8 (80)	2 (67)

**Table 2:** Participant use and engagement in the program.

Variable	Participants, mean (SD)		Mentors, mean (SD)
	Experimental	Control	
Comfort with computers	2.58 (0.65)	2.46 (0.66)	—^a^
Comfort with discussion forum	1.54 (0.72)	1.62 (1.04)	—
Time spent on the website, hours	5.22 (4.95)	2.95 (2.47)	25.3 (22.3)
Number of logins	7.17 (6.00)	7.66 (5.02)	72.3 (78.05)
Number of messages	8.58 (6.02)	8.11 (4.85)	65.6 (46.49)
Self-rated engagement	6.47 (2.40)	5.56 (3.53)	7.25 (0.27)

^a^Not applicable.

### Time Spent Online

The experimental group spent more time online (mean 5.22, SD 4.95) compared with the control group (mean 2.95, SD 2.47) and posted slightly more (experimental: mean 8.58, SD 6.02; controls: mean 8.11, SD 4.85), although the difference was not significant (see Table 2). Meanwhile, the mentors spent an average of 25.3 (SD 22.3) hours online, mean of 72.5 (SD 78.05) logins to the website, and mean 65.6 (SD 6.49) posts. Both groups logged in a similar average amount (experimental: mean 7.17, SD 6.00; controls: mean 7.66, SD 5.02).

### Recruitment

We sent information packages via mail or email to 812 potential participations. A researcher followed up with participants via phone or email about 1 week after initial contact to assess for potential interest in the study. Youth who were interested in taking part were screened for eligibility, and if interested, they signed a written consent form. We reached and assessed 309 participants for eligibility. A total of 503 potential participants were unable to be contacted to participate in the study (see Figure 1 for reasons). Moreover, 37 participants were randomized (24 in the 3 experimental groups and 13 in the 2 control groups) and 28 completed the intervention or control and the postsurvey.

### Engagement and Satisfaction With the Intervention

The experimental group reported a higher self-rated engagement (mean 6.44, SD 2.33) compared with the control group (mean 5.56, SD 3.53), although the difference was not significant. There was a significant difference between the experimental group reporting that they would recommend the program to others compared with fewer of the controls 100%, (18/18) experimental and 78% (7/9) controls (*P*=.04). In the open-ended survey comments, participants described what they liked most about the program, including the career and life-skills content, shared experiences with mentors and other participants, and the format of the intervention (see Table 3).

### Secondary Outcome Analysis

Parameters for our secondary outcome analysis explored recruitment and accrual rates, satisfaction with the intervention, and suggestions for improvement. Marginal models were computed to examine group differences on each outcome. No significant differences on outcome measures were noted at baseline. After controlling for baseline scores, no significant differences were found in career maturity, self-determination, or social support between the experimental and control groups after the intervention (see Table 4 for the comparison of secondary outcome measures between the 2 groups).

**Table 3:** Overview of themes and representative quotes. Quotes are provided in italics, the Participant ID is in brackets.

Theme	Experimental group	Control group
Engagement and satisfaction	*This is a topic I have to start thinking about...it initiated some good conversations between my mother and me.* [#1-04]; *It made me think about the things that are important for finding a job.* [#2-08]	*This program prepares you for how to find work and shows the different steps.* [#5-02]; *It covers things that school doesn’t.* [#3-07]
Connecting and learning from others	*The responses from the mentors made me feel what I had to say meant something. It was so neat to learn from other’s experiences and how I could relate.* [#1-01]; *(I liked) how there were other clients with a disability struggling with the same thing I am in terms of job searching that I can sort of relate to.* [#2-02]; *The different aspects and questions that were presented made me think about problem-solving skills...you don’t feel alone and you get different viewpoints from others.* [#2-04]; *As someone who is preparing themselves for work, I found that reading the strategies that were provided by the other participants in the study group was very helpful to me, as I can utilize them and apply them to my own job search.* [#1-03]	*My favorite part of the program was reading other participants responses.* [#4-06]
Module content	*Most topics were very thought provoking which allowed me to develop thought-out personal answers.* [#1-02]	*Being able to read other posts and see the differences and similarities. I enjoyed the modules (power points) with useful knowledge.* [#4-03]; *I feel like the program did a good job of teaching how to get a career with a disability.* [#3-07]
Areas for improvement	*The website was challenging to use.* [#5-03]	*More people on at once and face-to-face interactions.* [#5-03]; *If there were more interaction and discussion, it would be more useful.* [#4-06]; *I already struggle with communicating to strangers so I was reluctant to comment on others posts.* [#4-03]; *I just hope next time the website works better...whenever I tried to log in I had a problem with my password.* [#5-01]

**Table 4:** Descriptive statistics on participant outcomes by treatment condition.

Variable	Pretreatment, mean (SD)		Posttreatment, mean (SD)		Test statistic *F (df)*	*P* value	Effect size (Cohen *d*)
	Experimental	Control	Experimental	Control			
Self-determination	17.76 (8.67)	22.77 (7.59)	17.94 (7.33)	25.50 (9.31)	3.01 (27)	.97	0.9
Career maturity	13.93 (4.59)	15.00 (4.27)	14.11 (4.55)	15.57 (4.57)	0.256 (27)	.61	0.32
Social support	56.82 (8.04)	55.22 (14.92)	51.82 (16.24)	59.22 (13.84)	2.10 (27)	.16	0.49

## Discussion

### Principal Findings

Improving the employment preparation skills of young people with disabilities is important for enhancing their inclusion in the workforce. Youth with disabilities continue to experience higher unemployment rates compared with youth without disabilities [7,45]. Consistent research shows that mentoring can help to improve academic and employment outcomes [13]. Offering peer mentorship through an accessible format may help to provide social support to youth with disabilities while engaging them to learn about employment preparation skills [6,13]. Our study addresses an important gap in the literature by offering a Web-based, group-based peer mentoring intervention to help youth with physical disabilities prepare for employment. Many youth with physical disabilities have difficulty accessing vocational training. A virtual learning environment can offer a space for participants to access key resources [46].

Our results indicate that the *Empowering youth towards employment* intervention was feasible and acceptable to the participants. Our findings show that the majority of participants were satisfied with the intervention and would recommend it to other youth. In the qualitative feedback, participants reported that the program was beneficial for employment preparation and interacting with other youth that have a disability, especially those in the experimental groups. Participants in the control groups reported satisfaction with content and employment readiness. These results are encouraging and help to show that there is a need for the content. Few participants within the control group described social interactions or sharing experiences with other youth, which could be because of the lack of a mentor to facilitate conversation. We also observed that among the few participants that posted in the control group, there was very little interaction between the participants. Previous studies show that having a moderator in a discussion forum can help participants to feel at ease while also providing information [46]. Given that both the experimental and control groups reported valuing the support provided, further work is needed to fully tease apart how much these sessions were valued because of the mentor’s presence or because of the contact with and shared information with peers and overall social support.

The findings revealed that the self-reported engagement in the study was somewhat lower than that expected for participants in both the control and experimental groups. Other research similarly shows that maintaining engagement of participants in an e-mentoring study can be difficult [47,48]. The relatively low engagement in our study indicates that the intervention format and length might need adjustment and further testing. We decided to have a 4-week version of this e-mentoring intervention to account for youth’s busy schedules during the school year; thus, we ran our intervention in the summer months. Their low engagement levels could have been because of youth’s involvement in other activities and because they lacked time to participate fully in the program. Future studies should consider how best to keep youth engaged, perhaps through reminders or having a variety of different components (face-to-face, either in person or through *Skype,* and Web-based, etc), or embedded in another type of therapy or educational program. It may also be helpful to organize more synchronous, group-based discussions.

The findings of our secondary outcomes showed no significant differences between the experimental and control groups on self-efficacy, self-determination, or social support. These results differ from the outcomes of our 12-week version (ie, 1 weekly topic) of this intervention [6], where we found a significant improvement in self-determination in the experimental group compared with the controls. Although other studies on e-mentoring interventions for youth with disabilities have shown significant improvements in self-determination [20,49], very few of these studies had RCT or other rigorous designs [14]. Of the few studies that have had an RCT design, that is, Ammerlaan et al [49] explored the impact of their interactive, group-based website for youth with juvenile arthritis, they also found no significant differences between the experimental and control groups on self-efficacy, quality of life, or self-management. Therefore, further testing and RCTs are needed to better understand how we can best design (ie, format, length and content) an intervention for youth with physical disabilities. In altering the original format of our intervention from 12 weekly topics [38] to 3 topics per week over 4 weeks (ie, the study reported here), we hoped to increase engagement and potential outcomes; however, this was not the case. Although youth reported that they liked this format, there were no significant improvements in the outcome we measured. These results suggest that it may take time to see a change in social support, self-determination, and career maturity. Further research is needed to continue testing the optimal dosage and format of the intervention.

Our nonsignificant findings contrast some other studies using an e-mentor approach for youth with disabilities. For example, Bell [50] focused on youth with vision impairments and found significant improvements in career decision-making. The format of their intervention involved several components including face-to-face, group-based activities, email, and phone calls [50]. Meanwhile, Kim-Rupnow and Burgstahler [51] had a Web-based group mentoring program for youth with various types of disabilities and found significant improvements in career options, employment preparedness, perseverance, social skills, and self-advocacy. These previous studies suggest that several components are needed to help keep youth engaged in the program, an issue that was noted by the youth’s self-reported engagement and also in their postsurvey comments. Most other research using an e-mentor intervention for youth with disabilities do not have a control group [14] and might account for some of the nonsignificant findings. Our results could also reflect that youth need more time to develop their career decision-making skills, which is something that may not be captured within a 4-week time frame.

Although the qualitative feedback from participants was encouraging regarding the social support they received during the intervention, this was not sufficient for detecting a significant difference in outcomes between the experimental and control groups. These findings may be because of it being a pilot and having insufficient power to detect changes. It could also be partly a result of the format or length of the intervention. For example, some youth expressed that they wanted more group-based and synchronous chats, which could help to improve their engagement and overall feeling of support. Other research on e-mentoring for youth with disabilities shows that such a forum can offer solution-focused support and shared experiences [52]. Previous studies show that a limitation of electronic forms of mentoring include that such forms of communication lack verbal cues and can result in impersonal relationships [14].

### Limitations and Future Directions

There are several limitations within this study that are important to consider. First, given that this was a pilot feasibility RCT, the study may have been underpowered and we did not reach our original target sample size [6] that was recruited from 1 site, which limits the generalizability of the findings. Future studies should aim to recruit from more centers and also offer youth different timing options to maximize the sample size and power. We also recognize that the uneven distribution of experimental and control groups, which was because of a timing issue of recruitment (ie, during the summer months), could have affected the power and significance of the findings. We encountered issues with recruitment and had a low overall response rate. Future studies should consider recruiting through different mediums (ie, social media and Web-based forums), broadening the inclusion criteria (age range, disability type, and employment experience), and partnering with other organizations. Second, we were unable to establish how much time each participant spent reviewing the modules, which could influence their employment preparation skills, career maturity, and self-determination. Third, there were several technical difficulties with the website over the course of the study (ie, difficulties logging in, glitches because of website upgrades, etc). Fourth, there was staff turnover in the mentors who lead the discussion forum. Although they were provided the same training and had a similar level of experience, this could have affected outcomes. Fifth, we included various types of physical disabilities that differ in nature and how this could influence self-management of their condition [53] and youth employment. Future studies should consider focusing on a specific disability type. Finally, the dosage of the intervention varied depending on how much people logged in and may have influenced outcomes. Other studies on e-mentoring for youth with disabilities have involved email, virtual environments, *Skype* video calls, and phone calls. Future research could consider incorporating some of these formats [14]. A recent review of e-mentoring for youth with disabilities found that the majority of studies involved one-to-one mentoring and some had a combination of both one-to-one and group-based mentoring [14]. Thus, future studies should consider offering more than 1 approach to maximize youth’s engagement in the study. Further work is also needed to explore any potential gains from such interventions on employment over the longer term.

### Conclusions

In conclusion, the *Empowering youth towards employment* intervention demonstrated that it is feasible and acceptable to the youth participants; however, we found no significant improvements in social support, self-determination, or career maturity compared with the controls.

Further adaptations to the format (including various components) and length of the intervention are needed to increase the acceptability to larger proportions of youth with physical disabilities. An RCT with an adequate sample size is required to assess the overall effectiveness of the program.

## Appendix

Multimedia Appendix 1 CONSORT-eHEALTH checklist (V 1.6.1).

## Abbreviations

e-mentor: electronic mentor
e-mentoring: electronic mentoring
RCT: randomized controlled trial
